# FITC-Labeled RGD Peptides as Novel Contrast Agents for Functional Fluorescent Angiographic Detection of Retinal and Choroidal Neovascularization

**DOI:** 10.3390/cells12141902

**Published:** 2023-07-21

**Authors:** Seung Woo Choi, Hye Kyoung Hong, Jehwi Jeon, Ji Young Choi, Minah Kim, Pilhan Kim, Byung Chul Lee, Se Joon Woo

**Affiliations:** 1 Department of Ophthalmology, Seoul National University College of Medicine, Seoul National University Bundang Hospital, Seongnam 13620, Republic of Korea; 2 Graduate School of Medical Science and Engineering, Korea Advanced Institute of Science and Technology (KAIST), Daejeon 34141, Republic of Korea; 3 KAIST Institute for Health Science and Technology (KIHST), Korea Advanced Institute of Science and Technology (KAIST), Daejeon 34141, Republic of Korea; 4 Department of Nuclear Medicine, Seoul National University College of Medicine, Seoul National University Bundang Hospital, Seongnam 13620, Republic of Korea; 5 Bio-Max Institute, Seoul National University, Seoul 08826, Republic of Korea

**Keywords:** fundus fluorescent angiography, choroidal neovascularization, RGD peptides, contrast agents, age-related macular degeneration

## Abstract

The development of choroidal neovascularization (CNV) is a crucial factor in the pathophysiology and prognosis of exudative age-related macular degeneration (AMD). Therefore, the detection of CNV is essential for establishing an appropriate diagnosis and treatment plan. Current ophthalmic imaging techniques, such as fundus fluorescent angiography and optical coherence tomography, have limitations in accurately visualizing CNV lesions and expressing CNV activity, owing to issues such as excessive dye leakage with pooling and the inability to provide functional information. Here, using the arginine−glycine−aspartic acid (RGD) peptide’s affinity for integrin α_v_β_3_, which is expressed in the neovascular endothelial cells in ocular tissues, we propose the use of fluorescein isothiocyanate (FITC)-labeled RGD peptide as a novel dye for effective molecular imaging of CNV. FITC-labeled RGD peptides (FITC-RGD_2_), prepared by bioconjugation of one FITC molecule with two RGD peptides, demonstrated better visualization and precise localization of CNV lesions than conventional fluorescein dyes in laser-induced CNV rodent models, as assessed using various imaging techniques, including a commercially available clinical fundus camera (Optos). These results suggest that FITC-RGD_2_ can serve as an effective novel dye for the diagnosis of neovascular retinal diseases, including AMD, by enabling early detection and treatment of disease occurrence and recurrence after treatment.

## 1. Introduction

Age-related macular degeneration (AMD) is one of the leading causes of irreparable blindness, particularly in the elderly [1]. The prevalence of AMD is increasing markedly, with the global number of patients with AMD projected to be 288 million in 2040. In Europe, that figure will rise by approximately 15% in 2050 compared to 2015 [2,3]. AMD can be classified into two subtypes, non-exudative (dry) and exudative (wet) AMD, based on the presence or absence of choroidal neovascularization (CNV) [4]. Although wet AMD accounts for only 10% of all AMD patients, approximately 90% of AMD-related severe visual loss is due to the worsening of wet AMD, significantly affecting the quality of life [5,6]. In addition, since 30% of dry intermediate AMD cases can be converted to wet AMD over 10 years [7], timely therapeutic intervention through regular retinal check-ups is essential to maintain good vision.

The generation of CNV through abnormal vascular invasion of pathological choroidal vessels into the area of retinal pigment epithelium (RPE) and subretinal space is the most crucial factor in the pathogenesis of wet AMD. The imperfect, flawed choroidal vessels can lead to the development of subretinal exudation and hemorrhage, resulting in the deterioration of the retinal structure and visual impairment [1,8]. Since earlier detection of a CNV lesion before it grows larger is associated with favorable visual outcomes and visual acuity preservation after intravitreal injection of anti-vascular endothelial growth factor (VEGF), the current standard treatment for wet AMD, quick and accurate CNV detection, is one of the critical processes in establishing a proper diagnosis and treatment plan [9,10,11,12].

Fundus fluorescent angiography (FA) has long been the traditional criterion for CNV detection [13]. The leaky vascular nature of CNV allows for its visualization and localization through the leakage of fluorescein dye during the FA process [14]. Due to the inherent limitation imposed by relying on dye leakage for CNV detection, the actual presence and structure of CNV can be obscured by unexpected dye leakage, resulting in a decline in the diagnostic value of conventional FA [15,16]. Furthermore, conventional FA with insufficient dye leakage from CNV may not accurately delineate the margin and extent of CNV lesions according to the CNV type in wet AMD [17]. In diagnostic FA, the use of fluorescent dye as a contrast agent has not evolved over the past 50 years [18]; thus, a different approach may be required to improve the diagnostic value and versatility of FA.

Biological processes and pathological lesions at the cellular, subcellular, or molecular level can be accurately detected and visualized using molecular imaging, an advanced medical multidisciplinary technique [19,20]. CNV pathogenesis involves many biological molecules and compounds that are possible indicators of CNV, owing to their unique characteristics; thus, molecular imaging targeting these moieties may be advantageous for the early detection and appropriate treatment of CNV compared to conventional FAs. However, despite this potential diagnostic and therapeutic benefit, molecular imaging for CNV visualization in AMD has rarely been investigated.

Since devising efficient functional probes for target molecule-specific interactions is a crucial step in the development of molecular imaging [21], in the pathogenesis of CNV, the identification of a suitable interaction between the targeting moiety of the probe and the target molecule is required. Integrins, transmembrane receptor molecules, are widely involved in cellular adhesion, integrating the extracellular and intracellular environments by exerting integrin receptor-ligand interaction [22,23]. As the expression level of integrins, particularly that of integrin α_ν_β_3_, increases distinctively in the pathogenesis of CNV associated with wet AMD, three-amino arginine−glycine−aspartic acid (Arg-Gly-Asp, RGD) peptides, which exhibit a selective binding property to the integrin receptor, may be an attractive targeting moiety candidate for the detection and visualization of CNV in wet AMD [23,24,25]. Our previous study demonstrated that integrin-binding RGD peptide-conjugated probes could be utilized for CNV imaging using single-photon emission computed tomography (SPECT) [26]. However, SPECT imaging requires radioactive tracers and has a low spatial resolution for use in the ophthalmologic clinical field compared to the FA.

**Figure 1:**
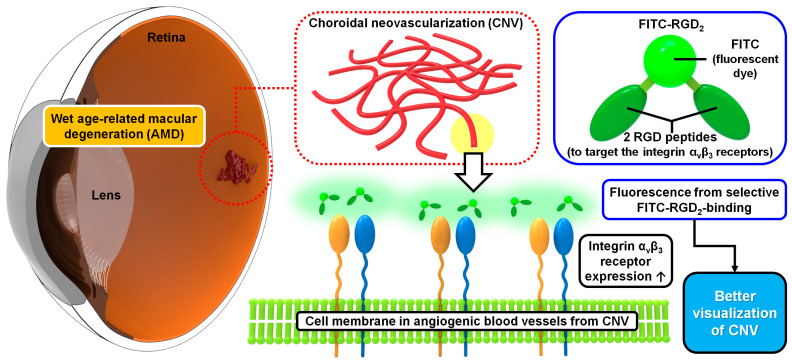
Schematics of the fluorescent angiographic detection of choroidal neovascularization (CNV) by FITC-labeled RGD peptides interacting with integrin receptors in CNV.

In this study, we designed fluorescein isothiocyanate (FITC)-labeled RGD peptides (FITC-RGD_2_) as novel fluorescent contrast agents based on in vivo molecular imaging (Figure 1 and Supplementary Materials, Figure S1) and investigated the diagnostic and structure-analytic potential of FITC-labeled RGD peptides using FA for CNV detection.

## 2. Materials and Methods

### 2.1. Methods

All commercial reagents and solvents were used without further purification. Common chemicals, solvents, and fluorescein isothiocyanate (FITC) were obtained from Merck (St. Louis, MO, USA), and Alexa Fluor™ 488 NHS Ester (AF488, Cat. No. A20000) was purchased from Thermo Fisher Scientific (Waltham, MA, USA). The cyclic peptide NH_2_-D-[c(RGDfK)]_2_ was prepared according to the procedures described in our previous study [26]. Electrospray mass spectrometry (ESI-MS) was performed using an LC/MS spectrometer (Agilent 6130 Series, Agilent Technologies, Santa Clara, CA, USA). The MiniTrap G-10 column was purchased from Merck (St. Louis, MO, USA).

### 2.2. Synthesis of AF488- and FITC-Labeled RGD Peptides (AF488-RGD_2_, FITC-RGD_2_)

FITC-D-[c(RGDfK)]_2_ (FITC-RGD_2_). NH_2_-D-[c(RGDfK)]_2_ (5.0 mg, 3.83 µmol) was dissolved in anhydrous DMF (1 mL). FITC (2.2 mg, 5.74 µmol) and diisopropylethylamine (1.33 µL, 7.66 µmol) were added to the solution. The reaction mixture was then stirred for 18 h at room temperature. After the conjugation was completed, the solvent was removed under reduced pressure. Purification was performed on a PD MiniTrap G-10 column to remove the unreacted fluorescent dye. The collected fraction was lyophilized, and a 73% yield of the product was obtained. MS (ESI) *m*/*z* = 1693.68 for [M + H]^+^.

AF488-D-[c(RGDfK)]_2_ (AF488-RGD_2_). Similarly, AF488-RGD_2_ (5 mg, 2.74 µmol) was prepared and purified according to the procedure described for FITC-RGD_2_. After purification by size exclusion column, the collected fraction was lyophilized, and the product was obtained with a 62% yield. MS (ESI) *m*/*z* = 1819.88 for [M + H]^+^.

Animal Information. All studies using a rodent model of CNV were approved by the Institutional Animal Care and Use Committee of Seoul National University Bundang Hospital (IACUC No. BA-2109-327-002-02) and adhered to the Association for Research in Vision and Ophthalmology (ARVO) statement for the Use of Animals in Ophthalmic and Vision Research. Wild-type 6-week-old C57BL/6 male mice (Orient Bio, Republic of Korea) weighing 22–25 g and Long-Evans male rats (Orient Bio) weighing 250 g were used for the experiments and validation, respectively.

Induction of CNV lesions. CNV lesions were induced in C57BL/6 mice and Long–Evans rats as described previously, with slight modifications [27]. A 512 nm argon laser system (Coherent PC-920 Argon Laser System; Coherent Medical Laser, Santa Clara, CA, USA) was used. Following intravenous anesthesia comprising a 1:1 mixture of 100 mg/mL ketamine and 20 mg/mL xylazine, and pupillary dilatation using 5.0% phenylephrine and 0.8% tropicamide, mice underwent laser treatment in the right eye. The laser delivery parameters were 100 mW power, 100 ms duration, and 100 μm of spot size. Five laser photocoagulation spots were used in each eye. A bubble that occurred immediately after the laser treatment was confirmed as successful laser induction. When massive subretinal hemorrhage occurred during laser treatment, we stopped the treatment and excluded the animals from the experiment. Fundus photography (Eyemera; IIScience, Republic of Korea) was used after laser treatment to verify the quality and location of the treated lesions. Choroidal flat-mount immunofluorescence staining was performed in the mouse model with FITC-RGD_2_ and lectin-BS1 using a previously reported method [26].

Induction of oxygen-induced retinopathy (OIR) mouse models. The OIR mouse model was established using a previously described method [28,29]. A mouse litter was exposed to 75% oxygen from postnatal day 7 (P7) to P12 (a total of 5 days) using a hyperoxia chamber. The mice were then returned to room air with normal oxygen levels to allow for the formation of new vessels. At P17, the mice were euthanized, and their eyeballs were retrieved for immunofluorescence staining. Following this, confocal microscopy was utilized to visualize CD31, integrin α_v_β_3_, Alexa Fluor 488-conjugated RGD peptides (AF488-RGD_2_), and DAPI, as previously described [29].

### 2.3. In Vivo Confocal Microscopic Imaging and Angiographic Evaluation in Mice

In vivo retinal imaging was performed using a custom-built laser-scanning confocal microscopy system modified for retinal imaging from a previously developed intravital confocal imaging platform [30,31,32]. Three continuous-wave laser sources, the 488 nm diode laser module (Cobolt MLD 488; HÜBNER Photonics, San Jose, CA, USA), 561 nm DPSS laser (Cobolt Jive; HÜBNER Photonics), and 640 nm diode laser module (Cobolt MLD 640; HÜBNER Photonics), were used as excitation light sources. A raster scanning pattern of the excitation laser was generated using a scanner system comprising a rotating polygonal mirror (MC-5; Lincoln Laser, Phoenix, AZ, USA) and a galvanometer-based scanning mirror (6230H; Cambridge Technology, Bedford, MA, USA), and then it was delivered to the back of the imaging lens aperture. A high-NA objective lens (PlanApoλ, 20X, numerical aperture = 0.75; Nikon, Tokyo, Japan) was used to provide a wide-angle fluorescence image of the retina. The fluorescence emission was detected using a multi-alkali photocathode photomultiplier tube (R9110; Hamamatsu Photonics, Hamamatsu, Shizuoka, Japan). The electric signal was digitized using a frame grabber (Solios; Matrox, Dorval, Quebec, Canada) and reconstructed to images with a size of 512 × 512 pixels per frame in real time. The anesthetized mice were placed on an articulating baseball stage (SL20; Thorlabs, Newton, NJ, USA) fixed to an XYZ translation stage (3DMS; Sutter Instruments, Novato, CA, USA). The maximum angle of view was previously measured as approximately 48°.

For in vivo retinal imaging, mice were anesthetized with a mixture of Zoletil (30 mg/kg) and xylazine (10 mg/kg) via intramuscular injection. The body temperature of the anesthetized mice was maintained at 36 °C using a homeothermic temperature monitoring and control system (RightTemp; Kent Scientific, Torrington, CT, USA) to prevent the abrupt formation of cold cataracts, which hamper the imaging of the retina. A xylazine antagonist, yohimbine (2 mg/kg), was injected to facilitate post-anesthesia recovery and stabilization of the cardiovascular system. Ophthalmic ointments and artificial tears were used to prevent corneal injury and dryness. 

**Figure 2:**
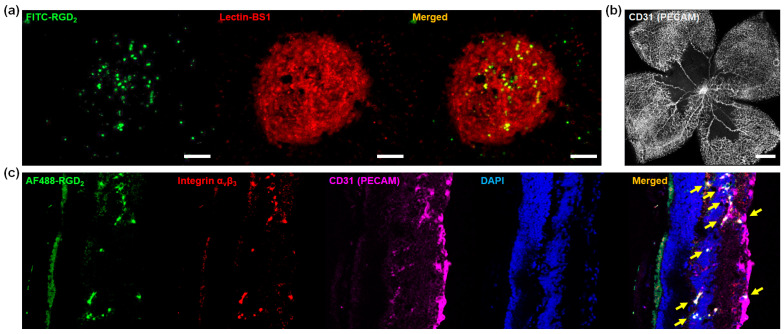
The results of immunofluorescence staining in the choroids of laser-induced choroidal neovascularization (CNV) mouse models and the retinas of oxygen-induced retinopathy (OIR) mouse models. (**a**) Colocalization of fluorescence signals from FITC-RGD_2_ and lectin-BS1 in the CNV lesions obtained 7 days after CNV induction with laser. (**b**) Representative retinal whole mounts of OIR mice at postnatal day 17 with CD31 (PECAM) fluorescence. (**c**) Immunofluorescence staining of retinal frozen sections obtained from OIR mice at P17. Colocalization of signals from CD31, integrin α_v_β_3_, and AF488-RGD_2_ is indicated by yellow arrows. Scale bars, 50 μm (**a**) and 500 μm for (**b**).

**Figure 3:**
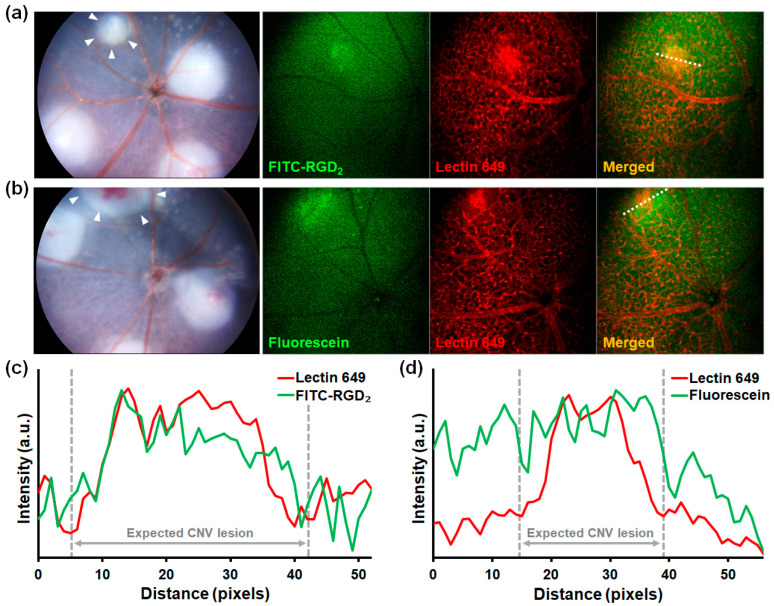
In vivo evaluation of localizing ability for CNV. A laser-induced CNV mouse model using C57BL/6 mice was used for these experiments. Representative fundus photographic and fluorescent angiographic images of the groups injected with (**a**) FITC-labeled RGD peptides (FITC-RGD_2_) and (**b**) fluorescein. Target CNV lesions were marked (white arrowhead) in the fundus photographs. (**c**,**d**) Comparison of fluorescent intensities of Lectin 649 (blood vessel marker) and fluorescent angiographic dyes in the vicinity of CNV lesions (white dotted lines) of each group measured using the plot profile function of ImageJ software (version 1.53t).

To visualize the CNV and CNV leakage, fluorescein sodium (Fluorescite Inj. 10%, Alcon, Geneva, Swiss, 240 μg, MW = 376 Da, 0.64 μmol in 100 μL of PBS) and FITC-RGD_2_ (1.35 mg, MW = 1692.4 Da, 0.80 μmol in 100 μL of PBS) were intravenously injected in each mouse (20 g) via tail vein catheters. Lectin DyLight 649 (DL-1178, Vector Laboratories, Burlingame, CA, USA, 3 mL/g) was intravenously injected to fluorescently label the vascular endothelial cells and delineate the vessel wall. Mice were injected simultaneously with fluorescent agents (fluorescein or FITC-RGD_2_) and lectin 649 at 7 days (Figure 2a) or 3 days (Figure 3) after the induction of CNV using an argon laser. Images were acquired around the CNV lesions 24 h after the injection of fluorescent agents. In vivo imaging experiments were independently conducted three times with the control group (average *n* = 5 per group). 

In vivo fluorescent angiographic ultrawide-field imaging in rats. FA was performed using a commercial fundus ultrawide-field (UWF) imaging system (Optos P200DTx; Optos PLC, Dunfermline, UK) in rat models following intravenous injections of 0.2 mL of 14 mg fluorescein sodium or 0.2 mL of 7 mg of FITC-RGD_2_ at day 3 after CNV induction with a laser. Angiographic images obtained using fluorescein sodium and FITC-RGD_2_ were analyzed and correlated with the fundus images. Unspecific leakage was defined as fluorescent leakage in areas that did not correlate with the location of the CNV lesions on fundus photographs. Fluorescein leakage from CNV lesions on FA was graded as previously described [33,34]. The extent of leakage was evaluated using the following scoring system: 0, faint hyperfluorescence or mottled fluorescence without any visible leakage (not leaky); 1, hyperfluorescent lesion without a progressive increase in size or intensity (suggesting possible leakage); 2, increasing hyperfluorescence intensity without any change in size (leaky); and 3, lesions with an increase in both hyperfluorescence intensity and size (significant leakage).

Statistical Analyses. Statistical analyses were performed using the SPSS software for Windows (ver. 21.0, SPSS Inc., Chicago, IL, USA). For nonparametric statistics, the Mann–Whitney U test was performed to determine the differences between the groups. Differences were considered statistically significant at *p* ≤ 0.05. 

## 3. Results

### 3.1. Characterization of the Interaction between Fluorescent Dye-Conjugated RGD Peptides and Integrins in Laser-Induced CNV Mouse Models and OIR Mouse Models

To evaluate the potential expandability to other neovascular eye diseases, fluorescent dye-conjugated RGD peptides were applied to laser-induced CNV and OIR mouse models. Seven days after CNV induction with a laser, immunofluorescence staining of the choroids showed effective colocalization of fluorescence signals from FITC-RGD_2_ with the fluorescent area of lectin-BS1, indicating successful targeting of FITC-RGD_2_ to the CNV lesions (Figure 2a). After OIR induction using mouse pups and hypoxic conditions, delayed retinal vasculature development was observed in the retinal whole mounts, as shown in the staining with platelet endothelial cell adhesion molecule-1 (PECAM or CD31), a surface marker of the constitutive cells for the vascular compartment (Figure 2b) [35,36]. The results of immunofluorescence staining using the frozen sections showed extensive neovascularization ranging from the internal limiting membrane to the inner nuclear layer in the OIR mouse models, visualized by CD31 fluorescence signal, and AF488-RGD_2_ exhibited a good targeting effect onto the integrin α_v_β_3_-expressed area which was part of the region that expressed CD31 (Figure 2c). In other words, since integrin α_v_β_3_ was expressed in some of the neovascular areas detected by CD31, dye-conjugated RGD peptides could visualize these areas by targeting the expression of integrin α_v_β_3_.

### 3.2. Comparison of CNV Visualization of Each Fluorescent Contrast Agent in the Laser-Induced CNV Mouse Models

The fluorescence angiographic results of both groups injected with FITC-RGD_2_ and fluorescein showed characteristic fluorescent signals in the vicinity of the CNV lesions (Figure 3a,b). However, compared to the accurate visualization of the CNV lesion by FITC-RGD_2_, the fluorescein dye demonstrated imprecise localization and fluorescent diffusion of the CNV lesion due to leakage. This obscuring effect caused by the leakage of the fluorescein dye was verified by measuring the fluorescence intensities of lines that passed through the CNV lesions (Figure 3c,d). The fluorescent signal of FITC-RGD_2_ was followed similarly to that of CNV lesions expressed by the fluorescence of lectin 649, which is used as an endothelial cell-specific marker. However, fluorescein dye without targeting ability showed high fluorescence intensities not only in CNV lesions but also in the SRF area around CNV lesions. 

### 3.3. Comparison of Ultrawide-Field Fluorescent Angiographic Results in Laser-Induced CNV Rat Models

**Figure 4:**
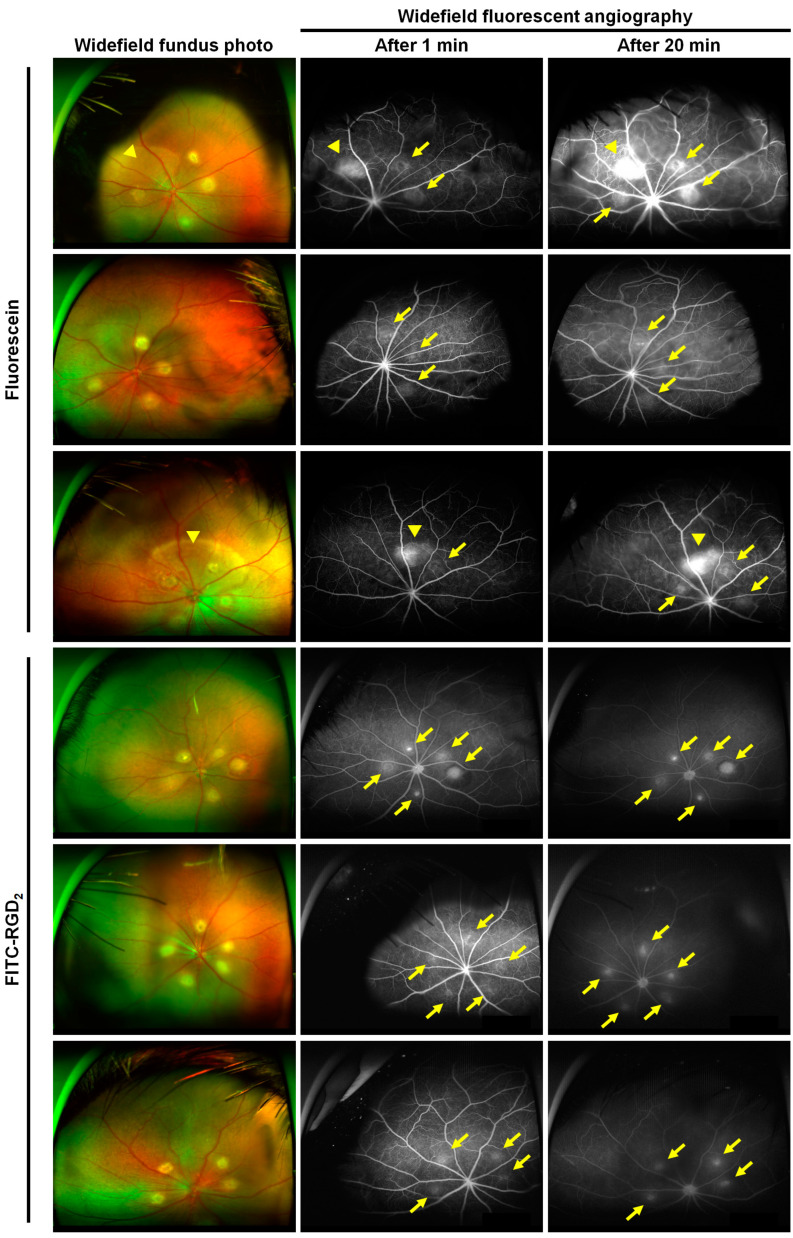
In vivo demonstration of CNV visualization through ultrawide-field fundus photography and fluorescent angiography with fluorescein and FITC-RGD_2_. A laser-induced CNV rat model using Long–Evans rats was used for these experiments. The angiographic images were obtained at 1 and 20 min after injection of each dye. A CNV lesion (yellow arrow) was visualized in groups injected with fluorescein and FITC-RGD_2_ (n in each group = 3), and the dye leakage irrelevant to CNV location (yellow arrowhead) was observed in the group injected with fluorescein.

In the group injected with FITC-RGD_2_, the fluorescence corresponding to the CNV lesion 1 min after dye injection appeared more distinctly and faster than that in the group injected with fluorescein. Further, the fluorescent signals became more pronounced in the late phase without margin blurring of the CNV lesions (Figure 4). The average grade of FA leakage was 2.0 ± 1.0 and 1.1 ± 0.7 for the groups injected with fluorescein and FITC-RGD_2_, respectively, indicating that FITC-RGD_2_ contributed to more distinct visualization of CNV lesions compared with fluorescein (Supplementary Materials, Figure S2). In addition, non-specific fluorescent signals irrelevant to laser-induced CNV location were observed in the group injected with fluorescein, and positive predictive values ([true positive / (true positive + false positive)] × 100) of the FA using fluorescein and FITC-RGD_2_ based on the images of fluorescent lesions were 81.8% and 100%, respectively, suggesting that the fluorescein dye may have a higher possibility of false positivity. Taken together, these results show that FITC-RGD_2_ is an efficient dye that can be used for the accurate diagnosis of active neovascular AMD in a commercial wide-field imaging system, without obscuring lesions due to dye leakage.

## 4. Discussion

In this study, we demonstrated the diagnostic potential of RGD peptide-conjugated fluorescent agents that target integrin α_v_β_3_ of retinal and choroidal neovascularization. In laser-induced CNV mouse and rat models, both FITC-RGD_2_ and fluorescein dye were detected using a laboratory animal apparatus as well as a commercial clinical fundus camera, showing CNV visualization through fluorescent expression, even though the administered amounts of FITC-RGD_2_ and fluorescein were not equal in the animal experiments (Figure 3 and Figure 4). Based on a recent study, it has been reported that the use of a reduced amount of fluorescein dye with the UWF imaging system (Optos) does not significantly affect the quality of FA images, including dye leakage and microvasculature [37]. Therefore, the dye concentration effect on the FA images in evaluating CNV is not expected to be significant. However, CNV visualization differed between the FITC-RGD_2_ and fluorescein dye. CNV is attributed to the formation of aberrant new vessels that exhibit vascular hyperpermeability owing to immaturity and reduced structural stability, resulting in fluid leakage and exudation [38,39]. By using the leaky characteristics of CNV, FA can be used to detect CNV and determine disease activity in ophthalmic clinics. As 40 kDa fluorescein-labeled dextran has been reported to exhibit an angiographic leakage from CNV and retinal vasculatures in cynomolgus monkeys and mice [40,41], FITC-RGD_2_ and fluorescein dye with molecular weights of approximately 1.7 kDa and 0.4 kDa, respectively [42], are both able to leak out and enable the visualization of CNV lesions. Moreover, due to its capacity to target CNV expressing integrin α_v_β_3_, FITC-RGD_2_ more clearly displayed the location and border of CNV lesions without blurring at both the early and late phases compared with the nonspecific pooling of fluorescein dye. Although dye leakage in FA can provide information regarding the disease activity of CNV lesions, it obstructs the actual position and structural details of CNV lesions [15]. Retinal diseases without CNV, such as central serous chorioretinopathy and macular edema, typically show leakage on FA; thus, leakage alone is insufficient to diagnose CNV. Indocyanine green angiography (ICGA), another widely used imaging modality in ophthalmology, can visualize CNV better than conventional FA because of the reduction in dye leakage attributed to the formation of larger ICG-protein complexes by the reversible binding of ICG to plasma proteins (particularly albumin). However, due to the low resolution of ICGA and the high cost of the fundus camera to detect the ICG fluorescence, ICGA is not feasible, especially in Western countries [27,43,44]. In addition, the CNV composed of loosely distributed vessels is difficult to visualize as ICG dye utilizes a cumulative effect to exhibit its function, and the ICG leakage can occur at the early phase of CNV formation, which can consequentially obfuscate the CNV structure [45]. In comparison, the FITC-RGD_2_ functions by displaying the interaction between the RGD moiety in FITC-RGD_2_ and integrin α_v_β_3_ in CNV lesions (which is more expressed at the early, active stage); thus, providing a clear visualization of CNV lesions, at both the early and active stage and filling in the information gap between FA and ICGA. Therefore, in addition to serving as a diagnostic tool for CNV, FA using FITC-RGD_2_ can also be used to evaluate angiogenic activity, facilitating the early prediction of disease recurrence and determination of optimal treatment timing. Optical coherence tomography (OCT) and OCT angiography (OCTA) are essential and useful imaging modalities for managing patients with AMD by visualizing retinal and CNV structures; however, they cannot provide functional information that expresses CNV activity [15,46]. As such, FA using FITC-RGD_2_ can be considered an alternative and complementary method for visualizing CNV and evaluating its activity simultaneously.

Visualizing detailed structures and assessing anatomical changes in CNV lesions is a key aspect of laser-induced CNV animal models, enabling efficient and valuable preclinical AMD research [27]. Although FA is one of the useful imaging techniques to estimate the contents of CNV in animal experiments, it is prone to unexpected, extensive dye leakage and pooling with worsening disease severity and older subject age [47,48]. These disadvantages of FA are attributed to the inherent characteristic of FA to determine CNV by dye leakage; therefore, FITC-RGD_2_, which uses target activity to visualize CNVs, might assist in analyzing the results of fundus angiography in animal experiments without extensive dye leakage. In addition, as the incidence of subretinal fluid, which can lead to nonspecific dye leakage, is higher in older than in young mice after CNV induction using a laser [49], FITC-RGD_2_ may be advantageous in this situation compared with the use of fluorescein dye.

As demonstrated in our previous study, the RGD peptide could be integrated into SPECT probes for CNV visualization; however, the actual clinical application of RGD peptide-conjugated probe ([^99m^Tc]IDA-D-[c(RGDfK)]_2_) was hampered by its inherent limitations such as exposure to radiation and need for the equipment to detect radioactivity [26]. FITC-RGD_2_ was devised to overcome these drawbacks and improve feasibility in clinical application. FITC-RGD_2_ and fundus angiography using FITC-RGD_2_ could serve as alternatives to fluorescein dye and conventional FA, respectively, thereby bridging the gap between molecular imaging and clinical practice. Furthermore, the clinical application of FITC-RGD_2_ has the potential to be extended to chorioretinal vascular diseases associated with integrins in their pathogenesis, such as AMD, proliferative diabetic retinopathy, and retinal vein occlusion [23,50].

The OIR mouse model is a well-known and widely used preclinical animal model for assessing abnormal angiogenesis, especially retinal neovascularization, which mimics the phenotypes of neovascular diseases. In the OIR mouse model, the RGD peptide-conjugated dye (AF488-RGD_2_) was able to selectively detect regions of high integrin α_v_β_3_ expression, which were portions of the area where CD31 was expressed (Figure 2). CD31, which serves as a marker for vascular endothelial cells, is expressed in both normal blood vessels and neovascular areas of OIR mouse models, with higher levels of expression observed in OIR retinas [51,52]. In contrast, integrin α_v_β_3_ is typically expressed in areas with neovascularization rather than in normal vessels, and its expression is significantly upregulated in the neovascular endothelial cells present in the retinas of OIR mice [53,54,55]. Therefore, visualization of the areas colocalized with CD31 and integrin α_v_β_3_ using AF488-RGD_2_ in our study, demonstrated that dye-conjugated RGD peptides could be utilized for the precise detection of neovascularization. In addition, CD31 also appears in various stages of CNV, including active, mid, and late stages, whereas integrin α_v_β_3_ is mainly expressed in the early, active stages of CNV [23,56]. Given these facts, RGD peptide-binding dyes may help visualize active pathologic CNV using their targeting ability as well as early detection of CNV generation, which is meaningful for preserving better visual outcomes in anti-VEGF treatment [9,57]. These findings suggest that the proposed fluorescent dye-conjugated RGD peptides (including FITC-RGD_2_) can be employed for the efficient detection of not only CNV but retinal neovascularization, expanding their usage to other eye diseases, including retinopathy of prematurity (ROP) and proliferative diabetic retinopathy, and AMD.

Safety issues are also important in real-world clinical applications. Previous studies have reported that FITC-conjugate, such as a FITC-dextran, was safe to use as a fluorescent dye in cynomolgus monkeys and human patients [40,58]. In addition, numerous studies have demonstrated the favorable safety and efficacy of RGD peptide-conjugated drugs and PET tracers, including ^18^F-labeled RGD-containing glycopeptide and [^18^F]Fluciclatide, in both preclinical and clinical trials, and many contrast agents with RGD are actively being used in oncology clinics [59,60,61,62]. Thus, findings from these studies suggest provisional evidence supporting the safety of FITC-RGD_2_ as a contrast agent, thereby facilitating its translation into clinical applications for chorioretinal imaging.

## 5. Conclusions

Dye-labeled RGD peptides, including FITC-RGD_2_, have been developed by linking dye molecules to RGD peptides. FITC-RGD_2_ effectively visualized neovascular lesions in both the OIR mouse model and the laser-induced CNV rodent model through their selective interaction with integrin α_v_β_3_ expressed in the neovascularization. Furthermore, compared to conventional fluorescein dye, FITC-RGD_2_ not only enabled better visualization of the fine structures and extent of CNV lesions by reducing the obscuring effect caused by dye leakage but also allowed assessment of disease activity in CNV lesions by leveraging the characteristics of integrin α_v_β_3_ expression. These results demonstrate that the proposed FITC-RGD_2_ assay can serve as a valuable alternative tool for visualization and diagnosis of ocular neovascularization, particularly CNV lesions, and can aid in determining the proper timing of treatment and follow-up plans in clinical practice using conventional fundus cameras. 

## Data Availability

All data of this study are available within the paper.

## Supplementary Materials

The following supporting information can be downloaded at: https://www.mdpi.com/article/10.3390/cells12141902/s1, Figure S1: Molecular structure of the fluorescein isothiocyanate (FITC)-labeled RGD peptides (FITC-RGD_2_). Figure S2: Comparison of the average grade of fluorescent leakages of CNV lesions based on the images obtained from fundus fluorescent angiography (FAG). * *p* < 0.05.
