# Supplementary material for: FITC-Labeled RGD Peptides as Novel Contrast Agents for Functional Fluorescent Angiographic Detection of Retinal and Choroidal Neovascularization
Source: Cells. 2023 Jul 21;12(14):1902. doi: 10.3390/cells12141902 (PMC10377818; doi:10.3390/cells12141902)
Supplement: Supplementary file 1 [file cells-12-01902-s001.zip › cells-2503934-supplementary.pdf]

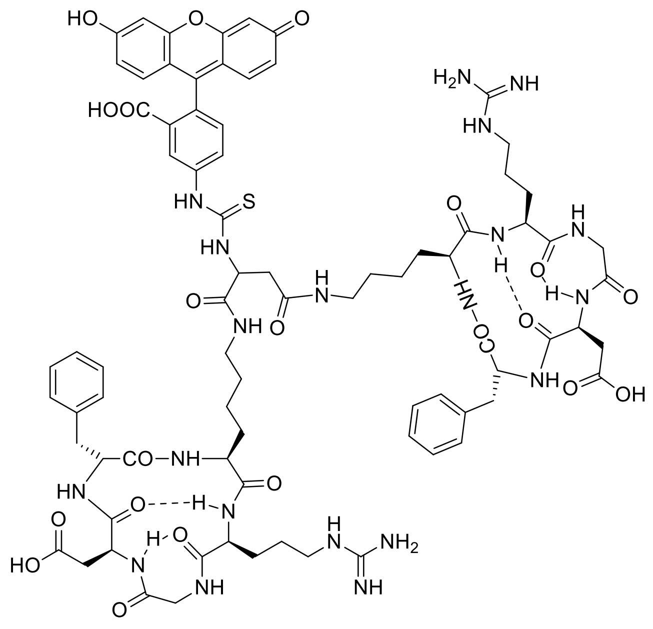

Figure S1. Molecular structure of the fluorescein isothiocyanate (FITC)-labeled RGD peptides (FITC-RGD2).

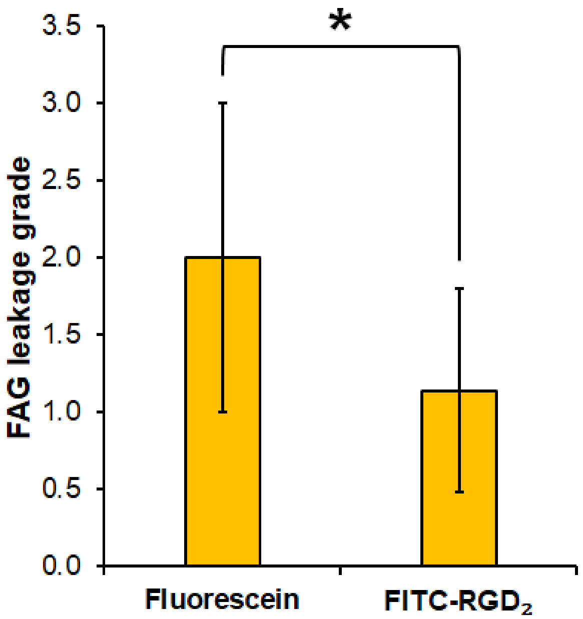

Figure S2. Comparison of the average grade of fluorescent leakages of CNV lesions based on the images obtained from fundus fluorescent angiography (FAG). *P < 0.05.
